# Economic assessment of the use of the sFlt-1/PlGF ratio test to predict preeclampsia in Germany

**DOI:** 10.1186/s12913-018-3406-1

**Published:** 2018-08-06

**Authors:** Dietmar Schlembach, Martin Hund, Annabel Schroer, Cyrill Wolf

**Affiliations:** 1 Vivantes Network of Health, Clinicum Neukoelln, Clinic of Obstetrics, Rudower Strasse 48, 12351 Berlin, Germany; 2 Roche Diagnostics International Ltd, Rotkreuz, Switzerland; 3 Roche Diagnostics Germany GmbH, Mannheim, Germany

**Keywords:** Angiogenic factors, Cost-effectiveness, Cost-saving, Diagnosis, Economic, Diagnosis-related group, Germany, Hospitalization, Hypertension, Preeclampsia, Pregnancy, sFlt-1/PlGF

## Abstract

**Background:**

The PRediction of short-term Outcome in preGNant wOmen with Suspected preeclampsIa Study (PROGNOSIS) demonstrated that a soluble fms-like tyrosine kinase 1/placental growth factor (sFlt-1/PlGF) ratio ≤ 38 ruled out the occurrence of preeclampsia in the next week with a negative predictive value of 99.3%; a ratio > 38 indicates an increased risk of developing preeclampsia in the next 4 weeks. We performed an assessment of the economic impact of the sFlt-1/PlGF ratio test for short-term prediction of preeclampsia in Germany.

**Methods:**

We adapted a cost-effectiveness model, which had been developed to estimate the incremental value of adding the sFlt-1/PlGF ratio test with a cut-off ratio of 38 to standard diagnostic procedures for guiding the management of women with suspected preeclampsia in the UK. We used the adapted model to estimate the incremental value of the sFlt-1/PlGF ratio test (cut-off 38) for guiding the management of women with suspected preeclampsia from a German Diagnosis-Related Group (DRG) payer perspective. The economic model estimated costs associated with diagnosis and management of preeclampsia in women managed in either a ‘no-test’ scenario in which clinical decisions are based on standard diagnostic procedures alone, or a ‘test’ scenario in which the sFlt-1/PlGF test is used in addition to standard diagnostic procedures. Test characteristics and rates of hospitalization were derived from patient-level data from PROGNOSIS. The main outcome measure from the economic model was the total cost per patient.

**Results:**

In the model adapted to the German DRG payer system, introduction of the sFlt-1/PlGF ratio test with a cut-off value of 38 could reduce the proportion of women hospitalized in Germany from 44.6 to 24.0%, resulting in an expected cost saving of €361 per patient.

**Conclusions:**

The sFlt-1/PlGF ratio test is likely to reduce unnecessary hospitalization of women with a low risk of developing preeclampsia, and identify those at high risk to ensure appropriate management. Even within the restrictions of the DRG system in Germany, this results in substantial cost savings for women with suspected preeclampsia.

**Electronic supplementary material:**

The online version of this article (10.1186/s12913-018-3406-1) contains supplementary material, which is available to authorized users.

## Background

Occurring in 6–8% of pregnancies and increasing, hypertensive disorders are the most common cause of maternal death in Europe, contributing to 20–25% of perinatal mortality [1, 2]. In higher income countries increases are attributed to rising obesity and advanced maternal age, trends that are likely to continue [2–8]. Preeclampsia, defined as new-onset hypertension and proteinuria or other maternal organ dysfunction, after 20 weeks of gestation [9], complicates approximately 2% of pregnancies [10–12] and is associated with substantial perinatal morbidity and mortality in mothers and infants [12].

Preeclampsia/eclampsia accounts for 10–15% of all maternal deaths (more than 70,000 maternal deaths per year worldwide) [12]. Long-term maternal complications of preeclampsia include an increased risk of cardiovascular disease, hypertension, ischemic heart disease, and stroke [13–16], as well as an increased risk of subsequent type 2 diabetes mellitus [16, 17], ophthalmological complications [18], end-stage renal disease [19, 20], and death [8]. Adverse outcomes are more common in future pregnancies in women who have experienced preeclampsia [21, 22].

More than 90% of maternal deaths from preeclampsia/eclampsia in Europe are potentially avoidable [23–25]. Failure to promptly recognize and treat hypertensive disorders of pregnancy, due to the variable clinical presentations and the low predictive value of high blood pressure and proteinuria, contributes to maternal mortality [2, 23, 26]. German Society of Gynecology and Obstetrics guidelines recommend that women with severe hypertension (≥ 160 mmHg systolic or ≥ 110 mmHg diastolic) or apparent preeclampsia are evaluated at the hospital, whereas mild pregnancy-induced hypertension is generally treated in the outpatient clinic [1]. However, diagnostic uncertainty means that women with suspected but not proven preeclampsia may be admitted to hospital unnecessarily, which can lead to substantial healthcare costs in addition to the costs of preeclampsia [27]. Thus, there is a need for rigorously evaluated tests that can predict or rule out preeclampsia with high sensitivity and specificity [28].

A diagnostic test, which uses the ratio of soluble fms-like tyrosine kinase 1 (sFlt-1) to placental growth factor (PlGF) to predict the short-term risk of developing preeclampsia [26], may help optimize patient management by triaging those at low risk of preeclampsia to an outpatient setting, while ensuring higher-risk patients are managed more intensively. PROGNOSIS (PRediction of short-term Outcome in preGNant wOmen with Suspected preeclampsIa Study), an international, prospective, observational study evaluated the use of sFlt-1/PlGF ratio, as determined by the Elecsys^®^ sFlt-1 and Elecsys^®^ PlGF assays, to predict the short-term (up to 4 weeks) risk of developing preeclampsia [26, 27]. PROGNOSIS derived and validated a cut-off value of 38 whereby: a sFlt-1/PlGF ratio ≤ 38 rules out the occurrence of preeclampsia in the next week with a negative predictive value (NPV) of 99.3% (and 97.9% for rule out within 2 weeks), and a ratio > 38 indicates that there is an increased risk of developing preeclampsia in the next 4 weeks [26, 29].

The economic impact of the test, based on PROGNOSIS data, has been assessed in the UK [30], and by the UK National Institute for Health and Care Excellence (NICE) to support their decision to include the sFlt-1/PlGF ratio in the new NICE guidelines for preeclampsia [31]. A further health economic assessment was performed in Italy [32]. However, such assessments cannot be extrapolated directly from one healthcare system to another and must be evaluated based on the unique system in each country. The payer system in Germany uses Diagnosis-Related Groups (DRGs) to assign a fixed payment for treatment of a specific condition. Therefore, the objective of this study was to assess the potential economic impact of using the Elecsys^®^ sFlt-1 and Elecsys^®^ PlGF assays for guiding the management of patients with suspected preeclampsia in Germany, and determine if use of the test remained cost-saving even within this DRG payer system. We adapted the cost-effectiveness model developed for the UK to allow us to evaluate the economic impact of the test from the perspective of the German healthcare system.

## Methods

### Model structure

#### Model development

**Fig. 1:**
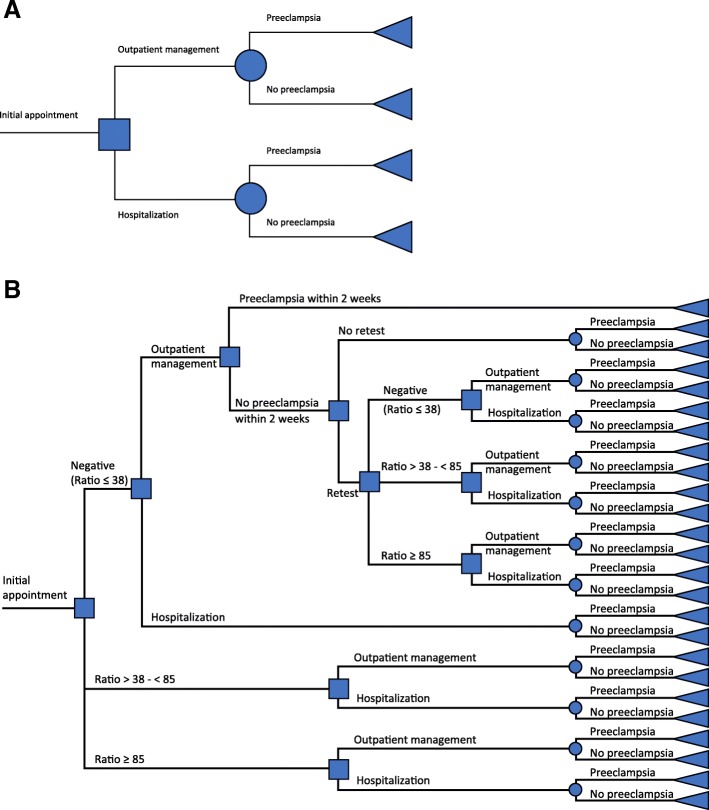
Decision trees in **a**) the no-test scenario, and **b**) the test scenario

An Excel-based cost-effectiveness model had been developed for the initial UK analysis, which we then adapted to allow for evaluation from a German payer perspective, in order to quantify the cost of managing suspected preeclampsia and/or hemolysis, elevated liver enzymes and low platelet count (HELLP) syndrome in German women [30]. At the time of analysis initiation, only one dataset was available with appropriate data, therefore a single-study approach was used. The original UK model and our updated German model both used data from the PROGNOSIS study [26, 27], and simulated the progression of a woman through a management pathway determined by the assessed risk of developing preeclampsia and the consequent decision to hospitalize or to manage the pregnancy in an outpatient setting with the focus on “ruling out” preeclampsia. A comparison of the expected cost of management was used to determine the incremental value of the results in ‘test’ (Fig. 1b: current diagnostic procedures including the sFlt-1/PlGF ratio) and ‘no-test’ (Fig. 1a: current diagnostic procedures without the sFlt-1/PlGF ratio) scenarios in all German women who met the inclusion criteria in PROGNOSIS (pregnant with clinical suspicion of preeclampsia in the absence of a definitive diagnosis). Key differences between the UK model and the German model were, briefly: that different patient management levels were applied according to the guidelines of each country; inputs such as cost data and hospitalization rate were specific to each country; the data input for the patient management decisions in the German model was derived from only the German population of PROGNOSIS. Full details of the differences between the UK and German models are provided in Additional file 1.

#### PROGNOSIS study data

Patient-level data from PROGNOSIS included hospitalization before developing preeclampsia, inpatient length of stay (LOS), the sFlt-1/PlGF test ratio, and diagnosis of preeclampsia. PROGNOSIS evaluated serum sFlt-1/PlGF ratios in 1050 women with suspected preeclampsia between 24 weeks and 36 weeks plus 6 days of gestation [26, 27]. The German Society of Gynecology and Obstetrics guideline was used as guidance for the patient management decisions applicable to Germany [1]. The aim of PROGNOSIS was to derive and validate a cut-off for the use of the sFlt-1/PlGF ratio in the short-term prediction of preeclampsia [26, 27]. During the study, decisions regarding the management of patients were made in the absence of serum ratio levels of sFlt-1/PlGF, due to unavailability [26, 27]. Fetal and maternal preeclampsia-related adverse outcomes, as well as resource use (e.g. planned/unplanned hospital admissions, LOS) were recorded [30]. Each participating study site of PROGNOSIS provided Ethics Committee/Institutional Review Board approval of the study protocol and associated documents (participant informed consent, participant information) before the start of the clinical part of the study. All women provided written informed consent before enrollment [26, 27]. Details of the study sites and Ethics Committee/Institutional Review Board approvals of PROGNOSIS are provided in Additional file 2. Ethics approval and participant consent for this health economics study was not necessary as it involved the use of a previously published de-identified database of PROGNOSIS according to German legislation.

#### Treatment scenarios

The patient treatment scenarios in this German model were specifically aligned with the German guidelines and the “Mutterschutzrichtlinien” (Maternity guidelines directives). Accordingly, the economic model included four different levels of intensity for management of hypertensive pregnancy disease: (1) outpatient management with regular measurement of blood pressure, determination of body weight, and assessment of proteinuria; (2) low intensity hospital management, with no longer than 1 day of hospitalized treatment; (3) intermediate intensity hospital management, with the period of hospitalization within the mean residence time (i.e. 2–9 days); and (4) high intensity hospital management, involving a period of hospitalization longer than the mean residence time (i.e. ≥ 10 days).

**Fig. 2:**
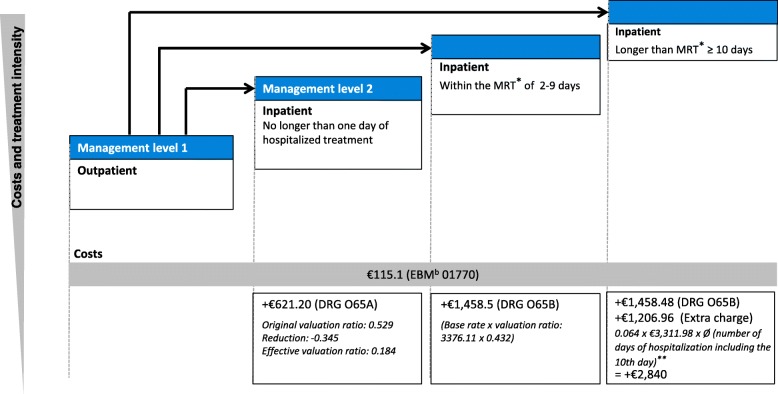
Preeclampsia management in Germany – four categories of treatment intensity (all costs are based on the EBM 2017 and DRG 2017). *MRT (2017): €3.376,11.**Ø = 15.2 days in PROGNOSIS. EBM, Einheitlicher Bewertungsmassstab; DRG, Diagnosis-Related Group; MRT, mean residence time

The percentages of women in each of the management intensity levels, based on PROGNOSIS data, were calculated as 55.4% in an outpatient setting (management level 1) and 44.6% in a hospitalized setting. Of the hospitalized women, 13.8% were treated in management level 2 (no longer than 1 day), 77.6% in management level 3 (2–9 days) and 8.6% in management level 4 (≥ 10 days) (Table 1; Fig. 2; Additional file 3). It should be noted that although the entire cohort of PROGNOSIS consisted of 1050 women, the population used in this economic analysis is based on the German cohort only (*n* = 204) in order to represent the German healthcare system as precisely as possible. The data input for the patient management decisions was derived from the German population of PROGNOSIS, whereas the data input for the prevalence of preeclampsia was derived from the entire population of PROGNOSIS.

**Table 1:** Management of women in Germany based on PROGNOSIS data

		Women, % (n)	
Management level 1	Outpatient	55.4% (113)	
Management level 2	≥ 1 day hospitalization	44.6% (91)	13.8% (12)
Management level 3	2–9 days hospitalization		77.6% (71)
Management level 4	≥ 10 days hospitalization		8.6% (8)

#### Classification by sFlt-1/PlGF ratio

Women were classified into three categories, specific to the German model, based on sFlt-1/PlGF ratio test results and the gestational week of pregnancy: ratio ≤ 38; ratio > 38 − < 85 (for gestational weeks 20 + 0–33 + 6) or > 38 − < 110 (gestational weeks 34 onwards); and ratio ≥ 85 (gestational weeks 20 + 0–33 + 6) or ≥ 110 (gestational weeks 34 onwards). The risk of preeclampsia and probability of hospitalization were assumed to positively correlate with the value of the ratio. The lower cut-off value of ≤ 38 to rule out preeclampsia within 1 week, with a NPV of 99.3%, was derived in PROGNOSIS [26, 27], and the higher cut-off value of 85 for the diagnosis of preeclampsia was derived in a multicenter case-control study [33, 34]. Percentages of women in each test result category were derived from the PROGNOSIS data. Analysis of the PROGNOSIS test scenario data revealed that 19.60% of participating women in the test scenario were shown to have a ratio ≥ 85 at weeks 20–33, or ≥ 110 from week 34 onwards. 16.20% of women had a ratio of > 38 – < 85 at weeks 20 + 0–33 + 6, or > 38 – < 110 from week 34 onwards, and 64.20% of women had a ratio ≤ 38. (Table 2).

**Table 2:** Assumed hospitalization rates in the no-test and test scenario (based on data in German women participating in the PROGNOSIS study)

	Distribution between categories	Assumed hospitalization rate
No-test scenario		44.6%
Test scenario		
sFlt-1/PlGF ratio ≥ 85 at weeks 20 + 0–33 + 6, or ≥ 110 from week 34 onwards	19.60%	70.0%
sFlt-1/PlGF ratio > 38 − < 85 at weeks 20 + 0–33 + 6, or > 38 − < 110 from week 34 onwards	16.20%	57.6%
sFlt-1/PlGF ratio ≤ 38	64.20%	1.5%

*Abbreviations:* PlGF, placental growth factor; sFlt-1, soluble fms-like tyrosine kinase 1

#### Clinical algorithm development

For the purposes of modeling the test scenario, a clinical algorithm was developed to estimate the disposition of women according to the value of the added ratio [1, 30, 35]. The assumption was that the proportion of women with a test ratio > 38 would be the same as that observed in the German cohort of PROGNOSIS [30]. During PROGNOSIS, test results were not available at the time of doctors’ patient management decisions, but were evaluated afterwards. Table 2 shows the hospitalization rates for the no-test and the assumed hospitalization rates for the test scenarios. Vatish et al. 2016 [30] stated that a ratio ≤ 38 would suggest a low risk of preeclampsia and, in principle, except for emergency situations, that no women would need to be hospitalized to manage the risk. Vatish et al. 2016 [30] continued to note that, in practice, there may be other reasons for hospitalization. Although there are potentially many different reasons that a clinician may make the assessment that a women with a ratio ≤ 38 may need to be hospitalized, unfortunately it was not possible to quantify all of these from the PROGNOSIS data. Thus, for pragmatic reasons, in the economic model we chose one example that was quantifiable from the available data, which was the assumption that a woman would be hospitalized if her blood pressure was higher than 160/110 mmHg. In the German population of PROGNOSIS, 1.5% of women met this blood pressure criterion and had a sFlt-1/PlGF ratio value ≤ 38 (Table 2). The economic model did not assume separate reimbursement for any particular treatments (e.g. corticosteroids, magnesium) given to women with a sFlt-1/PlGF ratio > 38, irrespective of hospitalization, since the German healthcare system is based on diagnostically related groups (i.e. clinics receive a set fee according to the diagnosis); healthcare costs remain the same within one diagnostically related group and the mean residence time irrespective of the treatment received.

#### Classification by sFlt-1/PlGF ratio after re-test

The economic model includes an option for a re-test 2 weeks after the initial test if the initial test was negative (ratio ≤ 38), they had been managed as an outpatient, and had not developed preeclampsia in the 2 weeks following their initial test. Women who were hospitalized were assumed not to be re-tested. The percentage of women who received a re-test was assumed to be 6.5%, which was derived from the Preeclampsia Open Study (PreOs; a multicenter, prospective, non-interventional study that examined the influence of sFlt-1/PlGF ratio on clinical decision making in the management of pregnant women with suspected preeclampsia in Germany and Austria [36, 37]). In PreOS, investigators were free in their clinical decisions, with no cutoff values recommended or clinical measures/procedures stipulated. However, investigators had access to the package insert for the test and were aware that an sFlt-1/PlGF ratio of ≥ 85 was useful for confirming a diagnosis of preeclampsia [33]. Percentages of women in each test category at re-test and the corresponding hospitalization rates were derived from PROGNOSIS data. During PROGNOSIS, test results were not available at the time of doctors’ patient management decisions, but were evaluated afterwards. Overall, 8.1% of women had a ratio of ≥ 85 at weeks 20 + 0–33 + 6 or a ratio ≥ 110 from week 34 onwards, of whom none were hospitalized; 13.5% of women had a ratio > 38 − < 85 at weeks 20 + 0–33 + 6 or a ratio of > 38 − < 110 from week 34 onwards, of whom 20.0% were hospitalized; and 78.4% of women had a ratio ≤ 38, of whom none were hospitalized.

### Cost analyses

In line with Vatish et al. 2016 [30], the cost analysis included the cost of the ratio test (€80), treatment cost of hospitalizations and outpatient appointments, anti-hypertensive medication and regular testing costs, the cost of preventing complications, and the cost of treating complications. Unit costs were taken from German-specific sources [38]. Final costs included a quarterly fee (€115.1) that is paid to outpatient doctors for every pregnant woman covering all examinations recommended by the “Gemeinsamer Bundesausschuss” (Federal Joint Committee) or the “Mutterschutzrichtlinien” (Maternity guidelines directives), multiplied by the average number of quarters (1.2) [39]. This was applied to all patients irrespective of whether they were hospitalized or not, in addition to the weighted total cost per hospitalization (applied to all hospitalized patients), which were based on the DRGs (DRG 2017) O65A and O65B and were calculated by multiplying the costs for low (assuming ≤ 1 day in hospital) (€621.2), intermediate (assuming 2–9 days in hospital) (€1458.5), and high intensity (assuming the average LOS of 15.2 days for hospital stays ≥ 10 days) management (€2840.0) by the proportion of patients managed in each of these settings.

### Sensitivity analyses

Sensitivity analyses were performed to test the robustness of the results. All scenarios used the German population of PROGNOSIS. The analyses consisted of: (a) variations in inpatient LOS, whereby LOS for hospitalized patients was increased and reduced by 20% (ranging from 12.2 to 18.2 days); (b) increment in hospitalization costs, whereby the average hospitalization costs were increased by 20% (from €1.462 to €1.754) due to the low reimbursement for hospitalization in Germany compared with the actual costs associated with the treatment and compared with other countries; and (c) variations in the proportion of women admitted to hospital depending on the value of the sFlt-1/PlGF ratio. For the latter, variations included a 5-percentage-point increase (from 1.5 to 6.5%) in the proportion of women hospitalized with a ratio of ≤ 38, and a 10-percentage-point variation in the proportion of women hospitalized with a ratio of > 38 − < 85 at weeks 20 + 0–33 + 6 or a ratio of > 38 − < 110 from week 34 onwards (ranging from 47.6 to 67.6%) or a ratio of ≥ 85 at weeks 20 + 0–33 + 6 or a ratio ≥ 110 from week 34 onwards (ranging from 60 to 80%). Furthermore, the effect of introducing a re-test has been analyzed in two different scenarios: (a) including a re-test rate of 6.5% derived from PreOS data for women treated in the outpatient setting who initially had a test result of ≤ 38 and did not develop preeclampsia at week two and (b) a re-test scenario where every woman was retested irrespective of whether she developed preeclampsia and irrespective of the initial sFlt-1/PlGF result.

## Results

### Cost analyses

The model shows that additional information provided by the sFlt-1/PlGF test ratio result in management decisions for women with suspected preeclampsia that are better correlated with preeclampsia outcomes than current diagnostic procedures alone. Without the test information 44.6% of women were hospitalized before a diagnosis of preeclampsia (Table 2), of whom 29.6% went on to develop preeclampsia (Table 3). If the additional information from the test had been available, the proportion of women hospitalized could have been reduced to around 24.0%, of whom 40.8% would have subsequently developed preeclampsia. This reduction in hospitalization is expected to generate a cost saving of €361 per patient (Table 3). The additional costs of the test and the re-test are more than offset by savings in the cost of hospitalization. It was shown that applying the test in a high-risk population not only reduces hospitalization by ruling out preeclampsia, but also ensures that more of the women who need hospitalization (i.e. women who will go on to develop preeclampsia) are hospitalized. Actually, the population of hospitalized women who developed preeclampsia increased from 29.6 to 40.8% (i.e. fewer false-positive results).

**Table 3:** Results of the cost analysis for the introduction of the sFlt-1/PlGF ratio test

	No test, n (%)	Test, n (%)	Difference, n
Patients hospitalized before preeclampsia, *n*	91	49	−42
Subsequently developed preeclampsia, *n* (%)	27 (29.6)	20 (40.8)	−7
Patients not hospitalized before preeclampsia, *n*	113	155	42
Subsequently developed preeclampsia, *n* (%)	19 (16.8)	26 (16.7)	7
Total cost, €			
Per cohort^a^	161,169	87,585	−73,584
Per patient	790	429	−361

^a^Total costs are shown for German participants of PROGNOSIS (*n* = 204 women)

### Sensitivity analyses

**Fig. 3:**
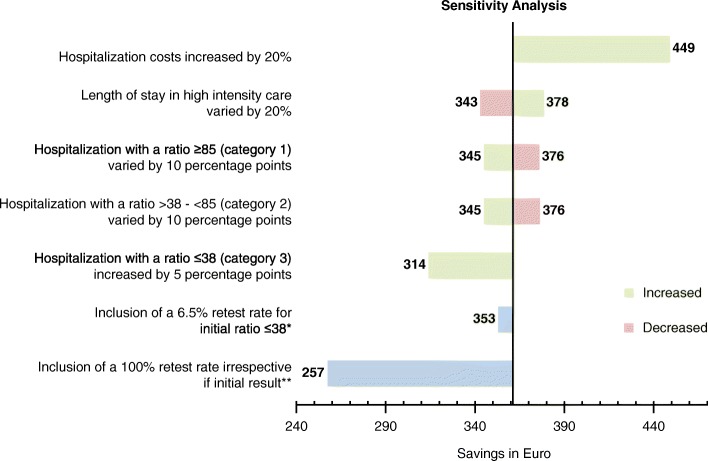
Results of sensitivity analyses. *Re-test scenario A: Re-test applied to 6.5% of women with initial ratio ≤ 38 who did not develop preeclampsia at week two and who were treated in an outpatient setting. **Re-test scenario B: Re-test applied to all women irrespective of the initial test result, preeclampsia status at week two and treatment location

The overall expectation of the positive value of the ratio test in terms of reducing costs is robust to plausible changes in the main parameters (Fig. 3). The greatest increase in cost savings could be shown by increasing the costs for hospitalization by 20%, which elevated the value from €361 to €449. Introducing a re-test for all women resulted in the lowest saving (€257). A 5-percentage-point increase in the proportion of women with a sFlt-1/PlGF ratio of ≤ 38 who were hospitalized resulted in a saving of €314. A variation of 20% in the average LOS for hospitalized patients resulted in savings ranging from €343 (LOS 12.2 days) to €378 (LOS 18.2 days). Varying the proportion of women being hospitalized with ratios of > 38 − < 85 or ≥ 85 at weeks 20 + 0–33 + 6, or ratios of > 38 − < 110 and ≥ 110 from week 34 onwards by 10 percentage points resulted in a saving ranging from €345 to €376. Introducing a re-test after 2 weeks of the initial test for 6.5% of all women with a ratio ≤ 38 who did not develop preeclampsia at week two and were treated in an outpatient setting (Table 4) resulted in a cost saving of €353.

**Table 4:** Criteria for the re-test scenarios

Re-test rate of 6.5%	Re-test for all women
Applied to all women with the following criteria: - initially had a test result of ≤ 38 - did not develop preeclampsia at week two - were treated in an outpatient setting The influence of the re-test for this population on the clinical and budget impact resulting from a change in management according to the re-test results and the additional test costs has been considered.	The influence of the additional costs on the budget impact for a re-test for all women irrespective of the initial test result, preeclampsia status at week two and treatment location has been taken into account. The influence of a re-test on the clinical impact resulting from a change in management according to the re-test results and the additional test costs has only been considered for all women with the following criteria: - initially had a test result of ≤ 38 - did not develop preeclampsia at week two - were treated in an outpatient setting

## Discussion

This economic analysis of patient-level data from the German cohort in the PROGNOSIS study demonstrates that even within the restrictions of this DRG payer system, use of the sFlt-1/PlGF ratio in women with suspected preeclampsia remains cost-saving, with an expected saving of €361 per patient. As for any such test, in order for payers to justify use of the test, country-specific health economics calculations are necessary to demonstrate the impact on healthcare budgets. The sFlt-1/PlGF ratio has previously been shown to improve the prediction of preeclampsia in women with suspected preeclampsia [26, 27] and, as a consequence, its use has been demonstrated to provide potential cost-savings for healthcare systems in the UK and Italy [30, 32]. Given that the annual number of live-born and stillbirths in Germany in 2015 was 740,362 [39], which we estimate to arise from approximately 726,450 pregnancies, and that approximately 15% of pregnant women in Germany develop signs or symptoms of preeclampsia [40, 41], the use of the sFlt-1/PlGF test has the potential to provide an annual cost saving in Germany of more than €39 million**.**

Our results are similar to those of an economic study, also based on data from PROGNOSIS, which assessed the impact of introducing the sFlt-1/PlGF ratio test into clinical practice in the UK [30]. Introduction of the test in the UK was estimated to reduce the number of women hospitalized by more than half (56%), from 36 to 16%, which was associated with a net cost saving of £344 in the base case analysis [30]. A similar study, in a cohort of 49,455 pregnant women in Italy based on patient-level data derived from PROGNOSIS reported expected net cost savings for the Italian National Health System of €671 per patient [32]. Although all three studies in Germany, the UK and Italy have shown cost-savings, lower cost savings demonstrated in our study in Germany compared with the Italian study are likely due to differences in the health systems in the respective countries; for example, differences in pricing and reimbursement for treatment of hypertensive women and general differences in patient management. The Germany payer system is based on a DRG rate per treatment of a specific disease, so the cost-savings we demonstrated directly reflect the reduced need for hospitalization, rather than cumulative reductions in use of individual interventions or resources for monitoring and treatment of preeclampsia.

Our results, which are based on real-life data, are more conservative than those reported in an earlier economic study from a German payer perspective, based on an assumed cohort of 1000 patients and published clinical data and expert opinion regarding German practice and resource utilization [42]. The study, which utilized a sFlt-1/PlGF ratio cut-off of 85 and assumed that the cost of the test was €34.40, with testing repeated up to three times at six-weekly intervals, demonstrated that introduction of the test was associated with cost savings of €637 per patient. These cost savings were mostly driven by a dramatic 71% reduction in the number of patients who would have been unnecessarily tested, treated and managed for preeclampsia under the standard practice scenario. Moreover, the fact that the study was based on private health insurance costs, which are higher than the public insurance costs used in our study, and that medication costs were based on different German DRGs, all contributed towards the differences in costs savings between this and our study.

A study by Schnettler and colleagues [43] included women before 34 weeks’ gestation with suspected preeclampsia. The study evaluated standard clinical assessment with and without sFlt-1/PlGF ratio (using a cut-off value of 85) from a US healthcare payer perspective. Base-case results show an overall cost reduction of $1215 per patient, from $3022 to $1807, associated with the use of the sFlt-1/PlGF ratio. Finally, a recent budget impact model using data from a prospective observational cohort study investigating the role of the PlGF test alone for triaging 625 women with suspected preeclampsia in the UK and Ireland, reported a mean cost saving associated with the PlGF test of £635 (95% CI − £1454 to −£4) per woman [44].

Within the German cohort of the PROGNOSIS study, 44.6% of women with suspected preeclampsia were admitted to hospital, the majority of whom did not subsequently develop preeclampsia (only 29.6% developed preeclampsia). We show that use of sFlt-1/PlGF ratio values of ≤ 38 would reduce the proportion of women hospitalized to 24.0%, which could potentially translate into a substantial reduction in burden on the German healthcare system. Indeed, the Elecsys sFlt-1/PlGF ratio test is already in clinical use in Germany using a cut-off value of 85 to “rule in” preeclampsia [1]. The multicenter, prospective, non-interventional PreOs study examined the influence of sFlt-1/PlGF ratio on clinical decision making in the management of pregnant women with suspected preeclampsia in Germany and Austria [36, 37]. Statistical analysis of outcomes was conducted using sFlt-1/PlGF categories ≤ 33, > 33 to < 85, and ≥ 85, with results from the study indicating that women at highest risk were correctly hospitalized after the sFlt-1/PlGF test, and that the risk for preeclampsia-related maternal and fetal outcomes increased along with increasing sFlt-1/PlGF ratios and was highest in women with an sFlt-1/PlGF ratio of ≥ 85 [37]. Among initially planned hospitalizations in PreOS, approximately one-third (32.5%) were identified as not being necessary in the short term. Results of our study support the results of the PreOs study and provide further evidence for use of the sFlt-1/PlGF test to support preeclampsia management in the German healthcare setting with the DRG payer system.

As with any study, our study has its strengths and limitations. A major strength is that the analysis was based on clinical data collected from a large observational study (PROGNOSIS) and, as such, is likely to reflect real-world clinical practice. Investigators in PROGNOSIS and the PreOS study were specialists in managing women with preeclampsia, therefore actual savings are likely to be higher than estimated here. This is because not all doctors in the real world are specialists for hypertensive disorders in pregnancy. Therefore, one would assume that hospital admissions in real-world clinical practice, especially unnecessary hospital admissions, are likely to be higher. Also, the scenario analyses indicated robustness of the base-case assumptions, with results shown to be sensitive to the hospitalization costs, LOS, and hospitalization rates with a negative test result (sFlt-1/PlGF ratio ≤ 38).

This study has limitations. The number of German women included in the study, and therefore included in our analysis, was relatively small. The overall savings found in this study in women with suspected preeclampsia may have been lower if a patient population with reduced risk, i.e. no clinical suspicion of preeclampsia, had been used in the model. A probabilistic analysis could have been performed to determine the probability that use of the sFlt-1/PlGF ratio remained cost-saving under the influence of uncertainty of the input parameters, however this analysis was not done; therefore, these data are not available. It is also conceded that data from a randomized controlled study, showing the actual impact of predicting preeclampsia with the sFlt-1/PlGF ratio test, are needed to confirm the value of this approach in clinical practice. Additionally, there are some restrictions associated with the analyzed re-test scenarios. The influence of the re-test results on patient management is only considered for the majority of women (including all women who initially had a test result of ≤ 38, did not develop preeclampsia at week two, and were treated in an outpatient setting). Additional test costs are considered for the entire cohort in the second re-test scenario (Table 4).

A major limitation of our study is that the real costs for preeclampsia in Germany are nearly impossible to calculate, as it is extremely difficult to get reliable data. To be as accurate as possible and only compare costs that arose from treating preeclampsia and exclude general care for pregnancies, only the DRGs for hospitalizations associated with preeclampsia that have not led to delivery have been considered (i.e. since the focus of our study was to “rule out” preeclampsia, we only consider the reduction in false-positive hospitalization not the possible additional correct-positive ones). The cost of treatment of women who developed preeclampsia in an outpatient setting could not be taken into consideration, even if they were hospitalized later as this would usually be associated with delivery. The clinical use of the sFlt-1/PlGF ratio for women with suspected preeclampsia would probably result in reduced admissions to neonatal intensive care, presumably by avoiding preterm deliveries. However, since costs for babies and mothers are not linked within the German healthcare system, neonatal costs could not be included in the model. Therefore, the costs used in our study are assumptions, and one would expect that in real life costs and associated savings might be higher.

Furthermore, long-term adverse outcomes in infants, irrespective of the effects of being born prematurely, have also been reported after preeclampsia, including epilepsy, autism, cardiovascular disease, stroke, and endocrine, nutritional and metabolic diseases in childhood [8, 45, 46]. Therefore, it is realistic, when considering the economic impact of preeclampsia, to not only consider costs associated with treatment of preeclampsia and the immediate adverse outcomes for both mother and child, but also those associated with long-term health consequences [8].

## Conclusions

Based on analysis of the German cohort of patients from the PROGNOSIS study, we demonstrate that use of sFlt-1/PlGF ratios of ≤ 38 is likely to reduce unnecessary hospitalization for women with suspected preeclampsia. We further demonstrate that this reduction in healthcare resource utilization translates into substantial cost savings for the German DRG payer system, supporting the sFlt-1/PlGF ratio test in women with suspected preeclampsia in Germany.

## Additional files

Additional file 1: Comparison of UK and German economic models. Key similarities and differences between the UK [1] and German economic models used to determine the incremental value of the sFlt-1/PlGF ratio test (cut-off 38) for guiding the management of women with suspected preeclampsia are presented. (DOCX 16 kb)

Additional file 2: PROGNOSIS study sites and Ethics Committee/Institutional Review Board approvals. Details of the study protocol approval at each of the PROGNOSIS study sites, including the site, Ethics Committee, Institutional Review Board approval number and final approval date. (DOCX 16 kb)

Additional file 3: Details of the assumed interventions according to intensity of patient management. Summary of interventions that *may* be performed under each management level according to German maternity policy guidelines and the S1-guideline for hypertensive pregnancy illnesses. (DOCX 16 kb)

## Abbreviations

DRG: Diagnosis-Related Groups
HELLP: hemolysis, elevated liver enzymes and low platelet count syndrome
LOS: length of stay
NICE: UK National Institute for Health and Care Excellence
NPV: negative predictive value
PlGF: placental growth factor
PreOs: Preeclampsia Open Study
PROGNOSIS: PRediction of short-term Outcome in preGNant wOmen with Suspected preeclampsIa Study
sFlt-1: fms-like tyrosine kinase 1
UK: United Kingdom
