# Supplementary material for: Economic assessment of the use of the sFlt-1/PlGF ratio test to predict preeclampsia in Germany
Source: BMC Health Serv Res. 2018 Aug 6;18:603. doi: 10.1186/s12913-018-3406-1 (PMC6080558; doi:10.1186/s12913-018-3406-1)
Supplement: Supplementary file 1 — Comparison of UK and German economic models. Key similarities and differences between the UK [1] and German economic models used to determine the incremental value of the sFlt-1/PlGF ratio test (cut-off 38) for guiding the management of women with suspected preeclampsia are presented. (DOCX 16 kb) [file 12913_2018_3406_MOESM1_ESM.docx]

**Additional file 1**

**Comparison of UK and German economic models**

Key similarities and differences between the UK [1] and German economic models used to determine the incremental value of the sFlt-1/PlGF ratio test (cut-off 38) for guiding the management of women with suspected preeclampsia are presented.

***Common features of the UK and German models***

Both decision-tree models had the same goal and the UK model [1] served as the template for the German model. They were developed to estimate the costs (inpatient/outpatient care) associated with diagnosis, monitoring and management of women with clinical suspicion of preeclampsia and/or HELLP syndrome to the country-specific healthcare systems. In both models:

- Costs included all preeclampsia/HELLP syndrome-related hospitalization and outpatient care from first presentation (at week 32) to preeclampsia, HELLP syndrome and/or birth.
- A full-term birth was assumed to occur at 40 weeks of gestation.
- Total costs for a cohort of women in a 'no-test' scenario (current standard of care) and a 'test' scenario (in which the new test is added to current standard of care) were compared.
- The criteria for the population included for the analysis were the same.
- The data input for the prevalence of preeclampsia was derived from the entire population of PROGNOSIS

***Differences between the UK and German models***

Due to fundamental differences in the health care systems, the German model was substantially adapted to the German healthcare system as follows:

- Although PROGNOSIS was the main database for both models, for the German model the data were filtered for only the German subpopulation to best represent the German healthcare system. Specifically, the data input for the patient management decisions in the German model was derived from the German population of PROGNOSIS, whereas the data input for the prevalence of preeclampsia was derived from the entire population of PROGNOSIS (as per the UK model)
- In contrast to the UK, German healthcare services are reimbursed via DRG payer system that include all the treatment and drugs costs for the treatment of a specific disease. In an outpatient setting this fee is usually paid for a whole quarter and in the hospital setting this fee is allocated to one diagnosis. In the hospital setting the reimbursement depends on the severity of a disease and on the length of stay (LOS), but there is not necessarily a linear relationship between costs and LOS. Therefore it was not possible to break down the costs to cost per day as it was done for the UK model; thus the structure of model was adjusted for use with German data.
- In the UK model, two ‘non-hospitalized’ categories and one ‘hospitalized’ category could be identified. In Germany, due to the reimbursement system and the German guidelines, four categories of treatment intensity (one outpatient and three inpatient categories) were identified (see Table 1, Figure 2 and Additional file 3), which are associated with different costs. Taking the German specific distribution between these scenarios from the PROGNOSIS database into account, the weighted average costs per patient hospitalized could be calculated.
- In contrast to the UK model, which did not consider weeks of pregnancy in the sFlt-1/PlGF ratio categories, the German model categorized the test results by gestational week, to match the German recommendations and/or clinical practice as closely as possible [2-5].
- In line with the UK model, it was assumed that a small amount of women would be hospitalized even though they have a test result <38 because they show signs and symptoms that should be assessed in a hospital (blood pressure ≥160 mmHg systolic or ≥110 mmHg diastolic). The guidelines in UK and Germany set the same threshold value, but in the German model the population who fit these criteria was adjusted to reflect the German population from the PROGNOSIS database (1.5 % for the German population).
- Finally, the re-test scenarios evaluated in the German model differ from the one used in the UK model. In the UK model, one re-test scenario was evaluated, in which the patient received another test after two weeks when the initial test was negative and when there were certain continuing symptoms manifesting. In the German model, two re-test scenarios were evaluated, in which the influence on the budget impact was calculated if the whole population received a re-test irrespective of the initial test result and one in which the percentage of the re-tested population was taken from the recent PreOs study [5].

References

1. Vatish M, Strunz-McKendry T, Hund M, Allegranza D, Wolf C, Smare C. sFlt-1/PlGF ratio test for pre-eclampsia: an economic assessment for the UK. Ultrasound Obstet Gynecol. 2016;48:765-771.
2. Dröge L, Herraìz I, Zeisler H, Schlembach D, Stepan H, Küssel L, Henrich W, Galindo A, Verlohren S. Maternal serum sFIt-1/PIGF ratio in twin pregnancies with and without pre-eclampsia in comparison with singleton pregnancies. Ultrasound Obstet Gynecol. 2015;45:286-283.
3. Verlohren S et al. New gestational phase−specific cutoff values for the use of the soluble fms-like tyrosine kinase-1/placental growth factor ratio as a diagnostic test for preeclampsia. Hypertension. 2014;63:346-352
4. Zeisler H, Llurba E, Chantraine F, Vatish M, Staff AC, Sennström M, Olovsson M, Brennecke SP, Stepan H, Allegranza D et al. Predictive value of the sFlt-1:PlGF ratio in women with suspected preeclampsia. N Engl J Med. 2016;374:13-22
5. Klein E, Schlembach D, Ramoni A, Langer E, Bahlmann F, Grill S, Schaffenrath H, van der Does R, Messinger D, Verhagen-Kamerbeek WD et al. Influence of the sFlt-1/PlGF ratio on clinical decision-making in women with suspected preeclampsia. PLoS One. 2016;11:e0156013.
