# Supplementary material for: Economic assessment of the use of the sFlt-1/PlGF ratio test to predict preeclampsia in Germany
Source: BMC Health Serv Res. 2018 Aug 6;18:603. doi: 10.1186/s12913-018-3406-1 (PMC6080558; doi:10.1186/s12913-018-3406-1)
Supplement: Supplementary file 2 — PROGNOSIS study sites and Ethics Committee/Institutional Review Board approvals. Details of the study protocol approval at each of the PROGNOSIS study sites, including the site, Ethics Committee, Institutional Review Board approval number and final approval date. (DOCX 16 kb) [file 12913_2018_3406_MOESM2_ESM.docx]

**Additional file 2**

**PROGNOSIS study sites and Ethics Committee/Institutional Review Board approvals**

| **Country** | **City** | **Institution** | **Ethics Committee** | **Institutional Review Board number** | **Final approval date** |
| --- | --- | --- | --- | --- | --- |
| Argentina | Buenos Aires | CEMIC | CEMIC | 772 | 30 August 2012 |
| Argentina | Buenos Aires | Sanatorio Guemes | FSG Fundación Sanatorio Güemes | Anmat N°6677/10; Salud N° 1490/07 | 26 September 2012 |
| Australia | Parkville, VIC | The Royal Women's Hospital | RWH Royal women´s Hospital | 12/32 | 23 November 2012 |
| Australia | Elizabeth Vale, SA | Lyell McEwin Hospital* | Government of South Australia Human Research Ethics Committee | HREC/12/TQEHLMH/71 | 16 January 2013 |
| Australia | Liverpool, NSW | Liverpool Hospital | NSW Government / Health Hunter New England Local Health District | HNEHREC Reference No: 12/07/18/4.01; NSW HREC Reference No: HREC/12HNE/224 | 12 October 2012 |
| Australia | South Brisbane, QLD | Mater Medical Research Institute | NSW Government / Health Hunter New England Local Health District | HNEHREC Reference No: 12/07/18/4.01; NSW HREC Reference No: HREC/12HNE/224 | 12 October 2012 |
| Australia | Richmond, VIC | Epworth Research Institute | Epworth HealthCare | 56112 | 9 November 2012 |
| Austria | Vienna | Medical University of Vienna, | Bundesamt für Sicherheit im Gesundheitswesen BASG/AGES | INS-621000-0225 | 30 March 2011 |
| Belgium | Liège | Centre Hospitalier Universitaire (CHU) de Liège | Comite D'ethique CHU | 1183 | 14 August 2012 |
| Canada | Montreal | CHUM (Centre Hospitalier de L’Universite de Montreal) | CHUM (University of Monterial hospital center) Research Ethics | 12.146 | 27 September 2012 |
| Canada | Burnaby | Pacific Centre for Reproductive Medicine, Canada | Schulman Associates IRB | 201205119 | 30 July 2012 |
| Canada | Halifax | IWK Health Centre | IWK-REB (Health Centre Research) | 1011968 | 16 October 2012 |
| Canada | Quebec | CHUQ-Pavillon CHUL | CHUQ University Hospital Center of Quebec | B12-08-1043 | 26 September 2012 |
| Chile | Osorno | Hospital Base Osorno | Ministerio de Salud Sevicio de Salud Valdivia Comité Etica de Investigación | N° 285 | 10 September 2012 |
| Chile | Concepcion | Hospital Regional de Concepcion | Comité Ético Cientifico del Servicio de Salud Concepción | 03948 | 7 November 2012 |
| Chile | Santiago | Instituto Chileno de Medicina Reproductiva | Scientific Ethics Committee Felix Bulnes Hospital Clinico | Acta N° 02/2012 | 5 September 2012 |
| Germany | Berlin | Campus Virchow-Clinic. Charitè University Medicine | LAGeSo Berlin | 10/0503 - ZS EK 11 | 13 September 2012 |
| Germany | Hannover | Hannover Medical School | Ethikkommission Medizinische Hochschule Hannover | 1385-2012 | 14 March 2012 |
| Netherlands | Maastricht | University Hospital Maastricht | Academisch Ziekenhuis Maastricht | 043-387 6009 | 23 May 2012 |
| New Zealand | Christchurch | Christchurch Hospital | Health and Disability Ethics Committees | CEN/12/06/029 | 20 August 2012 |
| New Zealand | Wellington | Wellington Hospital | Health and Disability Ethics Committees | CEN/12/06/029 | 20 August 2012 |
| Norway | Oslo | Oslo University Hospital | Regional komité for medisinsk og helsefaglig Forskningsetikk Oslo | 2010/2538 | 22 November 2010 |
| Peru | Surco | Instituto de Ginecologia y Reproduccion | PRISMA | CE1845.13 | 12 June 2013 |
| Peru | San Borja | Clinica Internacional Sede San Borja | PRISMA | CE1846.13 | 12 June 2013 |
| Peru | San Juan de Miraflores | Hospital Maria Auxiliadora | Hospital Maria Auxiliadora | 074 - 2012 - HMA - DOADI - CIEI | 11 October 2012 |
| Peru | Chiclayo | Hospital Regional Docente de las Mercedes | PRISMA | CE2373.12 | 13 September 2012 |
| Spain | Barcelona | Hospital Vall d´Hebron | Vall d´Hebron EC | n° 202 | 9 October 2012 |
| Sweden | Stockholm | Karolinska University Hospital | Regionala etikprövningsnämnden i Stockholm | 2011/151-31/3 | 23 February 2011 |
| Sweden | Uppsala | Upsalla Universtity | Regionala etikprövningsnämnden i Stockholm | 2011/1326 32 | 14 September 2011 |
| United Kingdom | Coventry | University Hospitals Coventry and Warwickshire | National Research Ethics Service | 11/NW/0542 | 16 January 2013 |
