# Supplementary material for: Economic assessment of the use of the sFlt-1/PlGF ratio test to predict preeclampsia in Germany
Source: BMC Health Serv Res. 2018 Aug 6;18:603. doi: 10.1186/s12913-018-3406-1 (PMC6080558; doi:10.1186/s12913-018-3406-1)
Supplement: Supplementary file 3 — Details of the assumed interventions according to intensity of patient management. Summary of interventions that may be performed under each management level according to German maternity policy guidelines and the S1-guideline for hypertensive pregnancy illnesses. (DOCX 16 kb) [file 12913_2018_3406_MOESM3_ESM.docx]

**Additional file 3**

**Details of the assumed interventions according to intensity of patient management**

**Management level 1 “Outpatient”:**

This includes interventions according to the German maternity policy guidelines and the suggested interventions according to the S1-guideline for hypertensive pregnancy illnesses. The S1-guideline states that mild gestational hypertension can be treated in the outpatient setting, with a weekly control-visit by the supervising gynecologist. Blood pressure, body weight and proteinuria should be measured. Fetal condition diagnostics including fetal growth, Doppler ultrasonography, cardiotocography and assessment of the amniotic fluid should be performed.

Also, laboratory tests should be performed to determine the following parameters:

- hemoglobin
- hematocrit
- platelets
- alanine transaminase (ALT)
- aspartate transaminase (AST)
- lactate dehydrogenase (LDH)
- bilirubin (indirect)
- urea
- creatinine
- proteinuria
- haptoglobin
- other blood coagulation tests (e.g. D-Dimer)
- PlGF (placental growth factor)
- sFlt-1/PlGF-ratio

**Management level 2 “Hospital management – low intensity”, Management level 3 “Hospital management – intermediate intensity” and Management level 4 “Hospital management – high intensity”:**

For all hospitalized management options the same recommendations are given; however, depending on the individual situation the frequency and intensity of interventions may differ.

At admission, condition diagnosis should be made to clarify if it is an emergency:

- Initial measurement of blood pressure at admission and ongoing strict measurement until stabilization
- Exclusion of prodromal symptoms (central symptoms, epigastric pain)
- Cardiotocography
- Proteinuria-diagnostic and quantitative protein determination
- Laboratory tests according to the list above

After stabilization:

- Blood pressure monitoring according to the clinical symptoms
- Cardiotocography (1–3 times a day)
- Laboratory tests as stated above, daily or twice a week (additionally determination of the sFlt-1/PlGF-ratio for short term prognosis and differential diagnosis)
- Monitoring of clinical symptoms, especially epigastric pain, headache, visual disorders, hyperreflexia
- Control of urine hourly in women with severe clinical symptoms of preeclampsia
- Pulse oximetry in women with respiratory symptoms
- Fetometry every 10–14 days
- Sonography of the fetus daily to weekly and assessment of the amniotic water
- Daily weight monitoring
- Prophylaxis of respiratory distress syndrome (RDS; weeks 24–34 of pregnancy) if necessary
- Medical therapy if necessary, only done in the hospital, e.g.:
  - Possible oral anti-hypertensives:
    - First choice: alpha methyldopa
    - Also possible: nifedipine, urapidel, dihydralazine
  - In case of pulmonary edema or cardiac insufficiency:
    - Furosemide, magnesium

Reference:

Stepan H, Kuse-Föhl S, Klockenbusch W, Rath W, Schauf B, Walther T, Schlembach D. Diagnosis and treatment of hypertensive pregnancy disorders. Guideline of DGGG (S1-level, AWMF Registry No. 015/018, December 2013). Geburtsh Frauenheilk. 2015;75:900-914.
